# Low-power laser therapy in chemical-induced oral mucositis: a case study

**DOI:** 10.5935/1808-8694.20130143

**Published:** 2015-10-08

**Authors:** Niedson José de Siqueira Medeiros, Nadson Frederico de Siqueira Medeiros, Carla Caroline Medeiros dos Santos, Georgia Veloso Ulisses Parente, Januse Nogueira de Carvalho

**Affiliations:** a Medical Student, Federal University of Campina Grande, UFCG (Medical Student); b MD, Graduated from the Federal University of Paraíba, UFPB (Physician of the Family Health Program (Tavares, PB), Attending Physician - Regional Hospital José Pereira Lima (Princesa Isabel, PB)); c Medical Student, Federal University of Campina Grande, UFCG (Medical Student); d Graduate in Dentistry, Pontifical Catholic University of Rio de Janeiro, PUC-RJ (DDS and dental expert and the Federal University of Campina Grande, UFCG); Federal University of Campina Grande, UFCG

**Keywords:** drug therapy, laser therapy, oral medicine

## INTRODUCTION

Oral mucositis (OM) is a common complication of chemotherapy and (or) radiotherapy, representing respectively, 40% and 100 % of cases 1., 2.. of oral mucosa inflammation1., 2. Erythema, ulceration, bleeding, swelling and pain are among the symptoms and signs, compromising nutrition, speech and fluid intake of the patients, predisposing them to systemic infection3., 4., 5.. The World Health Organization (WHO) classifies oral mucositis into: grade 0: absent; Grade 1: erythema, grade 2: erythematous and ulcerated, tolerating solids; Grade 3: erythematous and ulcerated, tolerating liquids only; Grade 4: erythematous and ulcerated, precluding feeding^3^. The low power laser therapy (LPL) works in the prevention and treatment of OM, providing for analgesic and anti-inflammatory effects, greater patient comfort, maintaining mucosal integrity and better tissue repair2., 3., 4., 5., 6.. With this paper we aim at analyzing the effectiveness of laser therapy in the treatment of oral mucositis.

## CASE PRESENTATION

**Figure 1:**
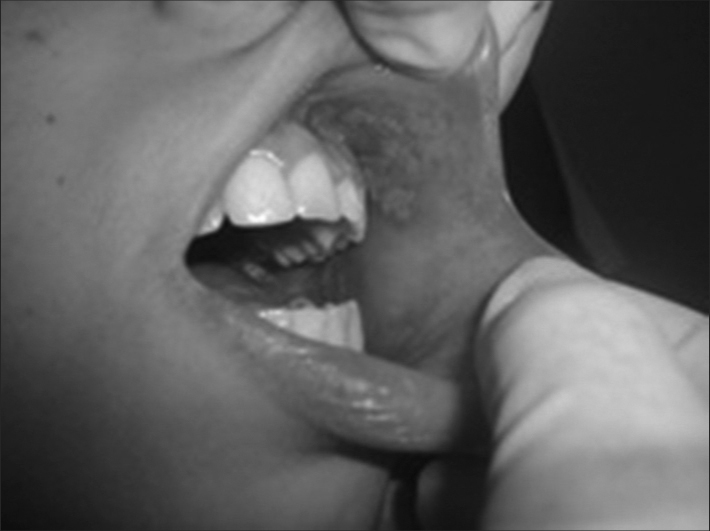
Region submitted to the laser therapy (660 nm wavelength, energy density of 4.3 J/cm^2^) - buccal mucosa.

BMCR - department of pediatric oncology, a 15-year-old female diagnosed initially with Ewing's sarcoma in the right clavicle, submitted to chemotherapy with ifosfamide, etoposide and vincristine (doxorubicin in subsequent cycles) under parenteral nutrition, complaining of intense pain. She had febrile neutropenia and pancytopenia, the patient received packed red blood cells and platelets and granulokine, cefepime, fluconazole and nystatin. Undergoing orthodontic treatment, with poor oral hygiene, she developed grade 3 mucositis lesions (WHO) in the buccal mucosa and left retromalar triangle. The dentist removed the device, removed the biofilm and polished her teeth. Laser therapy was instated three times a week for treatment of the mucositis lesions. We started with the 780 nm wavelength (λ) and an energy density of 4.3 J/cm^2^, and analgesic agent around the lesions. In the second session, we employed the therapeutic LPL at λ660 nm, at an energy density of 4.3 J/cm^2^, around the lesions (Figure 1). BMCR was instructed concerning oral hygiene and use of mouthwashes with chlorhexidine gluconate at 0.12%. After the first session, pain subsided and after the second, the patient was fed; after the fourth session, the lesions had decreased, healing almost entirely after the fifth application. A new cycle of chemotherapy was started two weeks later. Preventive laser at λ660 nm, energy density of 1.3 J/cm^2^ per point in the region of the buccal mucosa, mouth floor, tongue and palate, three times a week, was applied in order to avoid lesion recurrence. Currently, in the fourth cycle of chemotherapy, the patient no longer developed mucositis lesions. Thus proving the functional and clinical efficacy of the LPL: accelerating wound healing, reducing pain and length of hospital stay.

## DISCUSSION

Oral mucositis is defined as an inflammation and ulceration of the oral mucosa with pseudomembrane formation and potential source of infection, particularly febrile neutropenia^1^, such as it happened with our patient (BMCR). Pathologically, in mucositis there is a shallow ulcer generating interstitial exudate, cellular debris and fibrin, producing a pseudomembrane analogous to a superficial skin scar. The chemotherapy-induced mucositis varies from 40 % to 76 % for patients treated with standard and high-dose chemotherapy, respectively^1^. In intensive chemotherapy for relapse after remission, the association with ifosfamide, carboplatin and etoposide, and irinotecan are known to be toxic for the oral mucosal^2^. Antimetabolite agents (methotrexate, cytarabine, mercaptopurine), alkylating agents (melphalan busulphan), antibiotics (Doxorubicin) and etoposide, both used by the patient, also induce mucositis1., 2., 6.. In this case, the LPL eliminates pain already at the first application. This fact is attributed to the release of ß-endorphin in the nerve endings of the ulcer, while promoting tissue biostimulation, quickly repairing the ulcerations^2^.

## FINAL COMMENTS

It is necessary to encourage the use of low-power laser for the prevention and treatment of oral mucositis in cancer patients. It is a low cost and viable option in otorhinolaryngology, without side effects.
